# The pharmacokinetic study of tacrolimus and *Wuzhi* capsule in Chinese liver transplant patients

**DOI:** 10.3389/fphar.2022.956166

**Published:** 2022-09-15

**Authors:** Jinlong Qu, Rongrong Bian, Binguo Liu, Jiani Chen, Jingwen Zhai, Fei Teng, Wenyuan Guo, Hua Wei

**Affiliations:** ^1^ Department of Emergency and Critical Care, Second Affiliated Hospital of Naval Medical University, Shanghai, China; ^2^ Department of Nephrology, Kidney Institute of PLA, Second Affiliated Hospital of Naval Medical University, Shanghai, China; ^3^ Department of Pharmacy, Hospital of the Chinese People’s Liberation Army, Tianjin, China; ^4^ Medical Guarantee Center, Second Affiliated Hospital of Naval Medical University, Shanghai, China; ^5^ Institute of Organ Transplantation, Second Affiliated Hospital of Naval Medical University, Shanghai, China

**Keywords:** pharmacokinetic, tacrolimus, *Wuzhi* capsule, liver transplant patients, drug-drug interactions

## Abstract

**Objectives:** Wuzhi Capsule (WZC) is often administrated with tacrolimus in liver transplant patients to reduce the toxicity of tacrolimus and relieve the financial burden of patients. We aimed to investigate the interaction between *Wuzhi* Capsule (WZC) and tacrolimus in liver transplant patients.

**Methods:** We applied the LC-MS/MS analytical method previously established to study the pharmacokinetic characteristics of the analytes in 15 liver transplant patients. *CYP3A5* genotypes were determined in 15 donors and recipients, and they were categorized into *CYP3A5* expressers and non-expressers respectively.

**Results:** The influences of *CYP3A5* in donors and recipients on the pharmacokinetics of tacrolimus with or without WZC were also studied. We found that 1) WZC could influence the metabolism of tacrolimus, which shortened the Tmax of tacrolimus and decreased V/F and CL/F. 2) Moreover, our results showed that, in donors, the CL/F of tacrolimus were significantly lower in CYP3A5 (CYP3A5*1) expressers (decreased from 24.421 to 12.864) and non-expressers (decreased from 23.532 to 11.822) when co-administration with WZC. For recipients, the decreased trend of CL/F of tacrolimus was seen when co-administrated with WZC by 15.376 and 12.243 in CYP3A5 expressers and non-expressers, respectively.

**Conclusion:** In this study, the pharmacokinetics effects of WZC on tacrolimus were identified. The co-administration of WZC can increase the tacrolimus blood concentration in Chinese liver transplant patients in clinical practice.

## 1 Introduction

Tacrolimus, also known as FK506, is a calcineurin inhibitor (CNI), which is the cornerstone of immunosuppression after organ transplantation. Its strong immunosuppressive effect has made it the first choice for liver, heart, renal and bone marrow transplant patients in recent years. However, owing to its narrow therapeutic window, low oral bioavailability and large inter-individual variability in pharmacokinetics tacrolimus in many patients cannot reach the effective target blood concentration. Moreover, subtherapeutic tacrolimus concentrations are associated with an increased risk of allograft rejection (Huang et al., 2016), and supratherapeutic tacrolimus concentrations may cause adverse effects such as nephrotoxicity and neurotoxicity (Qin et al., 2014; Prytuła et al., 2019). Thus, routine therapeutic drug monitoring (TDM) for tacrolimus is recommended and seems to be increasingly important in adjusting the dose to improve the efficacy and tolerability of tacrolimus. TDM can facilitate clinicians to adjust drug dosage to ensure steady-state concentration of drug and avoid the occurrence of adverse reactions. Nonetheless, TDM still has some limitations, such as the disadvantages of complex detection methods and lagged detection results (Brunet et al., 2019).

*Wuzhi* Capsule (WZC), a preparation of an ethanol extract of Schisandra sphenanthera, is widely used in clinical practice in China to protect liver function in chronic hepatitis and liver dysfunction patients (Fan et al., 2015). Its main components including deoxyschizandrin, schisandrin, schisandrol, schisantherin and schisanhenol were identified and quantified. Several studies (Xin et al., 2011; Ye et al., 2016) have reported that WZC could significantly increase the blood concentration of tacrolimus in transplant patients to enable tacrolimus to reach a suitable blood concentration (Wang et al., 2016a),which was also seen at our clinical organ transplantation center. However, previous studies (Xin et al., 2007, 2011; Qin et al., 2014; Cheng et al., 2015) including our published articles (Xin et al., 2011; Wei et al., 2014) only determined blood concentration of tacrolimus or five bioactive ligands of WZC, or only studied the effects of the components of WZC on tacrolimus pharmacokinetics in transplant patients rats or healthy volunteers. There was no developed method in transplant patients to determine tacrolimus and five bioactive components of WZC simultaneously. Therefore, it is critical to study the interaction between tacrolimus and WZC.

Tacrolimus is primarily metabolized in the liver and small intestine by cytochrome P450 (CYP) 3A4 and 3A5 enzymes, and *CYP3A5* makes the major contribution to its pharmacokinetic changes (Cheng et al., 2015; Yousef et al., 2016). A polymorphism with high frequency within intron 3 (6986A>G, CYP3A5*3 allele) of *CYP3A5* gene, which is considered to be the primary mutation in Chinese, causes a splicing defect that leads to the absence of functional *CYP3A5* protein in homozygous carriers (CYP3A5*3/*3, *CYP3A5* non-expressers) (Chen et al., 2017). Only carriers of at least one CYP3A5*1 allele (wild-type allele) are classified as *CYP3A5* expressers. In the Han Chinese Population, the frequency of CYP3A5*1/*1,CYP3A5*1/*3 and CYP3A5*3/*3 is 8.4%, 34.3%, 57.3%, respectively. A series of studies (Argudo et al., 2015; Li and Li, 2015) have reported that higher dose-adjusted tacrolimus trough concentrations and lower tacrolimus dose requirements are shown in *CYP3A5* non-expressers (i.e., CYP3A5*3/*3) than *CYP3A5* expressers (i.e., CYP3A5*1/*1 or CYP3A5*1/*3). The results may rely on the fact that *CYP3A5* non-expressers (CYP3A5*3/*3) exhibit lower mRNA levels of *CYP3A5* expression. Therefore, the *CYP3A5* protein could not be expressed, which results in greater degradation of enzyme activity and lower metabolism of tacrolimus. Previous studies managed to minimize the potential distraction factors (Argudo et al., 2015) when studying the influence of *CYP3A5* genotype on the pharmacokinetics of tacrolimus, and most were conducted in renal transplant patients (Aouam et al., 2015; Cheng et al., 2015; Chen et al., 2017). However, we conducted our study from another perspective. What we did was to investigate the influence of *CYP3A5* genotype on the interaction of tacrolimus and WZC in liver transplant patients, in which the *CYP3A5* genotype of donors and recipients is urgent.

Thus, our aim of this study was to obtain accurate value of blood concentration at different time points to make pharmacokinetics study more reliable using on a LC-MS/MS analytical method for simultaneous determination of tacrolimus and five compounds of *Wuzhi* capsule; to investigate the pharmacokinetic characteristic of the analytes in liver transplant recipients’ whole blood in liver transplant patients based on this method; and to investigate the influence of *CYP3A5* genotype of donors and recipients on the pharmacokinetic characteristics of tacrolimus and WZC.

## 2 Materials and methods

### 2.1 Chemicals and reagents

The tacrolimus standard and Ascomycin (internal standard, IS) were purchased from Melone Pharmaceutical Co. Ltd. (Dalian, China). Deoxyschizandrin, schisandrin, and schisandrol B were purchased from National Institutes for Food and Drug Control (Beijing, China). Others contained schisantherin A provided by Shanghai Tauto Biotech Co.,Ltd., and schisanhenol provided by Beijing Beiyan Xinlv Biology technology Co.,Ltd.

HPLC grade methanol was provided by Merck Company (Darmstadt, Germany). All other reagents including Ammonium Acetate, and Zinc Sulfate were of analytical grade.

### 2.2 Instrumentation and chromatographic conditions

Experiments were carried out on an Agilent 1290 series high-performance liquid chromatography, including a G4220A quaternary pump, G4212A DAD detector and a G1316C column heater and a G4226A autosampler, a tandem Agilent 6460A triple-quadrupole mass spectrometer equipped with an electrospray ionization source (Agilent Inc., MA, United States) and operated with MassHunter Version B.06.00 workstation software. The separation was performed on an Agilent Zorbax SB-C18 column (3.5 μm, 2.1*100 mm) with the column temperature maintained at 55°C and the sample injection volume was 10 μl. The mobile phase consisted of a mixture of phase A (10 mM ammonium acetate buffer containing 0.1% formic acid) and phase B (methanol with 10 mM ammonium acetate buffer and 0.1% formic acid) with the flow rate at 0.25 mL min^−1^. The gradient program was set as follows: 0 min, 80% B; 0–2 min, 80–100% B; 2–10 min, 100% B.

Quantification was performed using electrospray in the positive mode with the spray voltage set at 4,000 V. Nitrogen was used as nebulizer gas and nebulizer pressure was set at 20 psi. Desolvation gas (nitrogen) was heated to 325°C and delivered at a flow rate of 12 L/min.

### 2.3 Whole blood sample pretreatment

The whole blood samples (100 μl) of liver transplant patients were pipetted into a microcentrifuge tube containing 10 μl IS solution (5 ng/mL). After vortexing the tube for 30 s, 200 μl ZnSO4 solution (0.1 mol/L) was added. After vortexing the mixture for 30 s, 300 μl methanol was added. After vortexing and mixing for 1 min and standing 10 min, the mixture was centrifuged at 15,000 r/min for 15 min. The supernatants of samples were used to inject into the HPLC system.

### 2.4 Preparation of solutions

The stock solutions of each analyte were prepared by dissolving them in methanol. The stock solutions except tacrolimus were mixed together to get the final concentration of 100 ng/mL for deoxyshisandrin, schisandrin, schisandrol B, schisanhenol, 600 ng/mL for schisantherinA. Working solutions were prepared freshly on each day of analysis as serial dilutions from stock solution by methanol. Each analyte was diluted to eight levels of concentration to construct the calibration curve: 0.2–60 ng/mL for tacrolimus, 0.10–100.00 ng/mL for deoxyshisandrin, schisandrin, schisandrol B, schisanhenol and 0.60–600.00 ng/mL for Schisantherin A, respectively. The internal standard (IS) stock solution was prepared by dissolving 2 mg of the Ascomycin in 1 mL of methanol. Then it was diluted to 5 ng/mL by methanol before use.

### 2.5 Method validation

The validation was performed to evaluate the performance of the method based on the recommendations published by the FDA (US Food and Drug Administration, 2013). See the detailed descriptions of these methods in Supplementary Material S1: Supplementary Method and our previous work (Wang et al., 2016b).

### 2.6 Pharmacokinetic experiments

#### 2.6.1 Subjects

A total of 15 patients were recruited among liver transplant candidates who first underwent liver allograft transplantation at Shanghai Changzheng Hospital from June 2014 to February 2015. Written informed consent was obtained from all recruited patients. This study was approved by Medical Ethics Committee of ChangZheng Hospital of Shanghai.

#### 2.6.2 Specimen collection and sample preparation

Postoperatively, the liver transplant recipients were initially treated with an immunosuppressive regimen based on tacrolimus. The initial dose of tacrolimus was 2 mg every 12 h. Then at the early stage of postoperation (within 30 days), WZC would be provided according to the actual situation of the patients. Dosage regimen was as follows: tacrolimus and WZC were administrated once every 12 h (at 6:00 and 18:00), of which the dose was 2 mg and 11.25 mg respectively. 2 mL of peripheral blood would be drawn into EDTA K2 anticoagulant tubes at 0, 0.5,1,2,4,8, and 12 h after administration of tacrolimus with WZC on the day of trial, which was stored at −20°C prior to analysis.

#### 2.6.3 Effects of *CYP3A5* genetic polymorphisms of donors and recipients on the pharmacokinetics of tacrolimus in liver transplant recipients

The blood samples were collected from the 15 donors and recipients. *CYP3A5* genotypes were determined by pyrosequencing and the donors and recipients were categorized as *CYP3A5* expressers (CYP3A5*1 allele carriers, Group A) and non-expressers (homozygous CYP3A5*3, Group B). The difference of pharmacokinetic parameters between group A and B was calculated under the following conditions: 1) when taking tacrolimus alone; 2) when taking tacrolimus and *Wuzhi* Capsule at the same time. And the pharmacokinetic parameters of tacrolimus before and after taking *Wuzhi* Capsule were also calculated in group A and group B, respectively.

### 2.7 Statistical analysis

Pharmacokinetic parameters were calculated using a noncompartmental analysis by the pharmacokinetic program (Data Access Service, DAS, version 3.2.7. Shanghai BioGuider Medicinal Technology Co., Ltd.). The pharmacokinetic parameters t1/2, Tmax, V/F, CL/F of tacrolimus before and after taking *Wuzhi* Capsule were compared by paired t-test. Covariance analysis was used to compare whether there is significant statistical difference of the C_max_ and C_0_ of tacrolimus with or without WZC.

## 3 Results

### 3.1 Method validation

The LC-MS/MS method in this study was previously developed and validated for simultaneous quantification of the tacrolimus and five bioactive lignan constituents (schisandrin, schisandrol B,schisantherin A, schisanhenol, and deoxyshisandrin) in WZC in liver transplant patients (Wang et al., 2016b). See the detailed results of the method validation in this study in Supplementary Material S2: Supplementary Result.

### 3.2 Pharmacokinetic experiments

#### 3.2.1 Characteristics of the study population and the frequency of *CYP3A5* variants

Of the 15 liver graft recipients, 11 were male and four were female. The mean age of patients was 51.72 ± 8.08 years. All patients were on tacrolimus-based immunosuppression in combination with *Wuzhi* Capsule. Of 15 donors, three were CYP3A5*1 homozygotes, six were CYP3A5*1/*3 heterozygotes, and six were CYP3A5*3 homozygotes, respectively. Of the 15 recipients, two were CYP3A5*1 homozygotes, seven were CYP3A5*1/*3 heterozygotes and six were CYP3A5*3 homozygotes.

#### 3.2.2 The pharmacokinetic parameters calculation of tacrolimus and main components of WZC in liver transplantation recipients

The pharmacokinetic parameters of all the analytes in liver transplantation recipients were calculated using a noncompartmental analysis by DAS (version 3.2.7) software.

**FIGURE 1:**
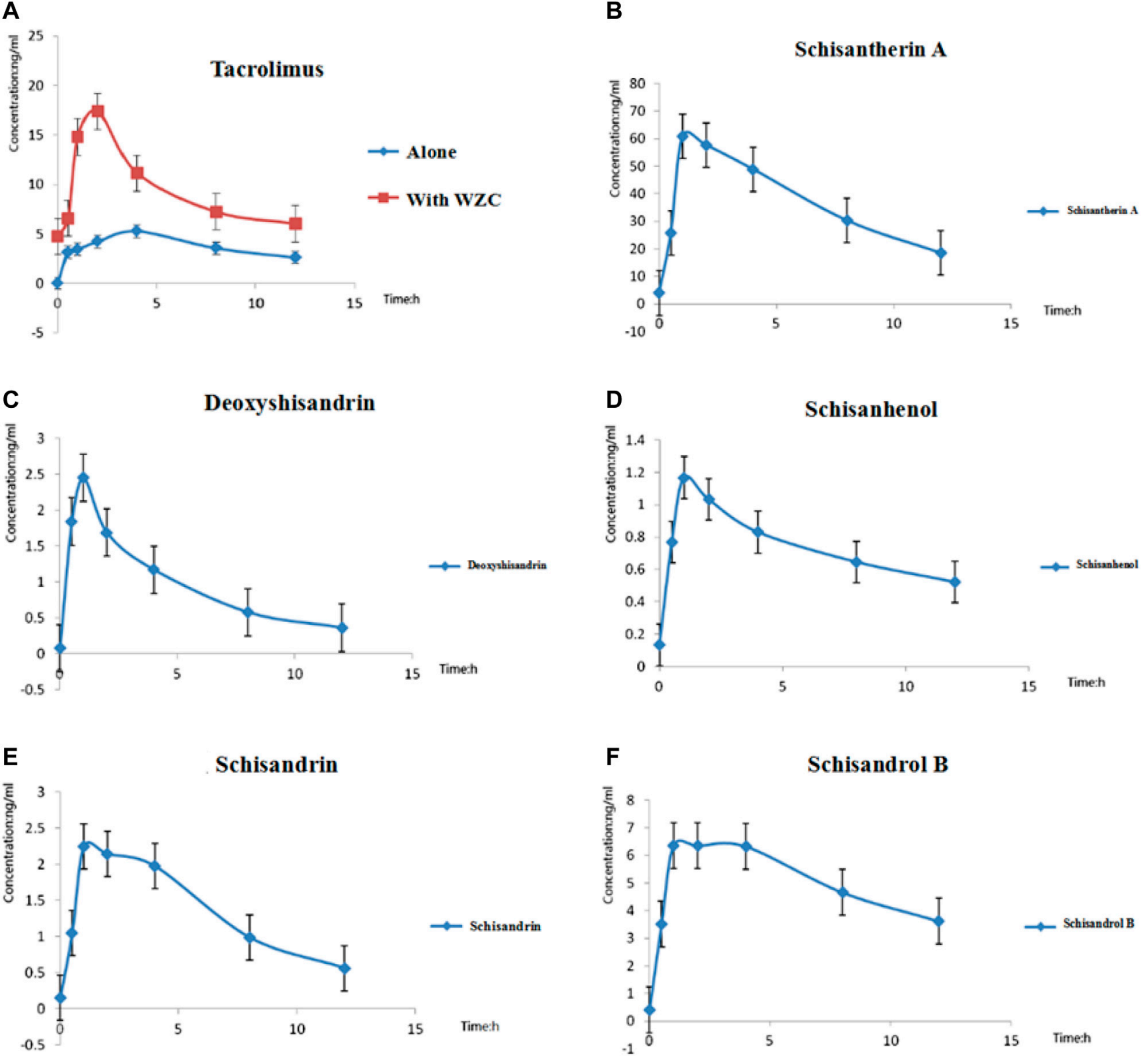
The blood concentration vs. time curves of all the analytes. **(A)** Mean blood concentration–time curves of tacrolimus with or without WZC (*n* = 15); **(B)** Mean blood concentration–time curves of schisantherin A after oral administration of tacrolimus with WZC (*n* = 15); **(C)** Mean blood concentration–time curves of deoxyshisandrin after oral administration of tacrolimus with WZC (*n* = 15); **(D)** Mean blood concentration–time curves of schisanhenol after oral administration of tacrolimus with WZC (*n* = 15); **(E)** Mean blood concentration–time curves of schisandrin after oral administration of tacrolimus with WZC; **(F)** Mean blood concentration–time curves of schisandrol B after oral administration of tacrolimus with WZC (*n* = 15).

First, the blood concentration at multiple time points in liver transplant patients after oral administration of tacrolimus with or without WZC was determined by LC-MS/MS described in “Materials and Methods”. The blood concentration vs. time curves of all the analytes were obtained and shown in Figure 1. And we also studied the pharmacokinetics of both tacrolimus and the five main components of WZC whose pharmacokinetic parameters were summarized in Table 1. We also compared the difference of tacrolimus pharmacokinetics parameters before and after taking WZC (shown in Table 2) and determined whether there was statistical difference between C_max_ and C_0_. The results showed that the pharmacokinetics of tacrolimus changed when liver transplant recipients co-administrated with WZC. As shown in Table 2, when tacrolimus co-administrated with WZC, Tmax was obviously decreased by 0.625 times (from 3.2 to 2.0 h). The result of Tmax indicated that the absorption of tacrolimus was accelerated with WZC. And both V/F and CL/F of tacrolimus were also decreased which had statistical variation. From Table 2, we could also find that the value of CL/F reduced from 23.935 L/h to 13.867 L/h (0.58 times lower than initial), which indicated that the WZC had a great influence on the clearance of tacrolimus in liver transplant recipients. However, there was no significant statistical difference between C_max_ and C_0_ when taking tacrolimus with or without WZC.

**TABLE 1:** The mean pharmacokinetic parameters of the analytes in liver transplant recipients (*n* = 15).

Parameters	Tacrolimus	Schisantherin A	Schisandrin	Schisandrol B	Deoxyshisandrin	Schisanhenol
C_max_ (ng/mL)	17.78 ± 11.92	58.50 ± 16.00	3.98 ± 2.52	10.79 ± 6.63	3.00 ± 2.56	1.25 ± 0.31
Tmax (h)	3.58 ± 3.32	2.50 ± 1.50	1.82 ± 0.65	1.64 ± 1.18	1.88 ± 1.35	1.36 ± 0.63
t_1/2_ (h)	16.59 ± 24.24	4.55 ± 1.78	4.00 ± 2.69	7.66 ± 2.27	5.13 ± 2.25	6.91 ± 1.98
MRT (h)	24.65 ± 34.38	6.09 ± 0.79	7.28 ± 4.91	11.44 ± 3.24	8.09 ± 4.42	11.23 ± 3.69
AUC_0-t_ (ng.h/mL)	112.87 ± 48.09	437.10 ± 251.41	16.17 ± 6.69	61.93 ± 23.50	11.75 ± 6.46	9.05 ± 2.97
AUC_0-∞_(ng.h/mL)	242.58 ± 114.91	619.10 ± 237.76	20.18 ± 6.08	93.59 ± 32.12	14.83 ± 6.81	15.59 ± 4.30

**TABLE 2:** The paired t-test result of pharmacokinetic parameters in liver transplant patients (*n* = 15).

Parameters	TAC alone (mean)	TAC + WZ (mean)	*p*
t1/2	11.673	8.702	0.319
Tmax	3.2	2	0.016^*^
Vz/F	388.969	135.632	0.031^*^
CLz/F	23.935	13.867	0.033^*^

*p < 0.05 significantly different as compared with tacrolimus alone group.

**FIGURE 2:**
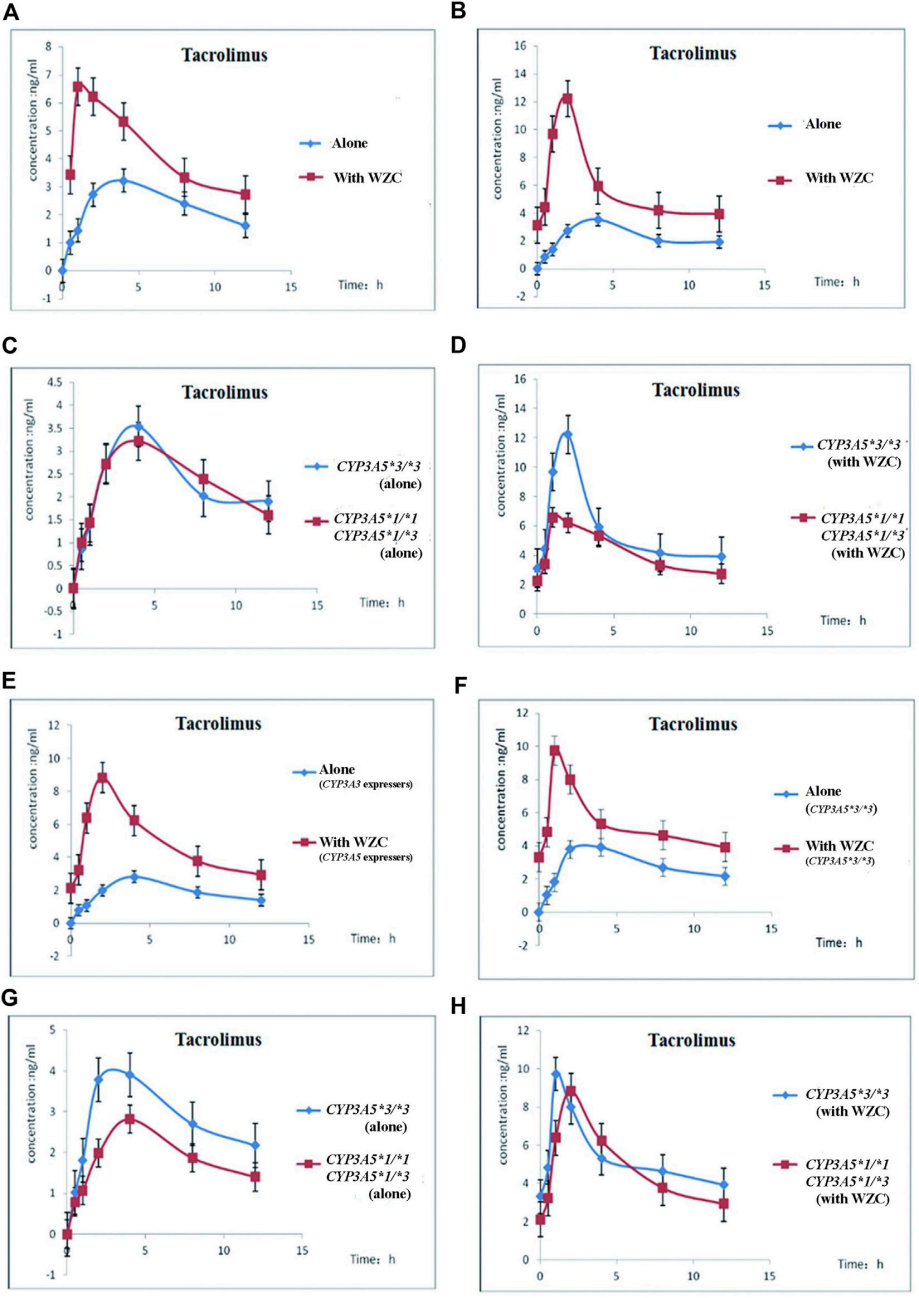
The blood concentration vs. time curves of tacrolimus in different CYP3A5 genotype donors and recipients before and after taking WZC. **(A)** Mean blood concentration–time curves of tacrolimus before and after taking WZC in CYP3A5*1/*1 or CYP3A5*1/*3 donors (*n* = 15); **(B)** Mean blood concentration–time curves of tacrolimus before and after taking WZC in CYP3A5*3/*3 donors (*n* = 15); **(C)** Mean blood concentration–time curves of tacrolimus between (CYP3A5*1/*1 or CYP3A5*1/*3) and CYP3A5*3/*3 donors when taking tacrolimus alone (*n* = 15); **(D)** Mean blood concentration–time curves of tacrolimus between (CYP3A5*1/*1 or CYP3A5*1/*3) and CYP3A5*3/*3 donors when taking tacrolimus combined with WZC (*n* = 15); **(E)** Mean blood concentration–time curves of tacrolimus before and after taking WZC in CYP3A5*1/*1 or CYP3A5*1/*3 recipients (*n* = 15); **(F)** Mean blood concentration–time curves of tacrolimus before and after taking WZC in CYP3A5*3/*3 recipients (*n* = 15); **(G)** Mean blood concentration–time curves of tacrolimus between (CYP3A5*1/*1 or CYP3A5*1/*3) and CYP3A5*3/*3 recipients (*n* = 15); **(H)** Mean blood concentration–time curves of tacrolimus between (CYP3A5*1/*1) or CYP3A5*1/*3) and CYP3A5*3/*3 recipients after taking WZC (*n* = 15).

Second, we compared the difference in tacrolimus pharmacokinetics parameters among different *CYP3A5* genotype donors and recipients before and after taking WZC. The comparison of blood concentration vs. time curves of tacrolimus were obtained under following conditions: 1) Before and after taking WZC in CYP3A5*1/*3 or CYP3A5*1/*1 donors (Figure 2A); 2) Before and after taking WZC in CYP3A5*3/*3 donors (Figure 2B); 3) Taking tacrolimus alone between CYP3A5*1/*3 or CYP3A5*1/*1 and CYP3A5*3/*3 donors (Figure 2C); 4)Taking tacrolimus with WZC between CYP3A5*1/*3 or CYP3A5*1/*1 and CYP3A5*3/*3 donors (Figure 2D); 5) Before and after taking WZC in CYP3A5*1/*3 or CYP3A5*1/*1 recipients (Figure 2E); 6) Before and after taking WZC in CYP3A5*3/*3 recipients (Figure 2F); 7) Taking tacrolimus alone between CYP3A5*1/*3 or CYP3A5*1/*1 and CYP3A5*3/*3 recipients (Figure 2G); 8) Taking tacrolimus with WZC between CYP3A5*1/*3 or CYP3A5*1/*1 and CYP3A5*3/*3 recipients (Figure 2H).When classified according to donors *CYP3A5* genotype (data shown in Table 3), the Tmax and CL/F of tacrolimus were significantly decreased in donors expressing *CYP3A5* (CYP3A5*1 carriers) after taking WZC, and there was significant statistical difference. When classified according to recipients *CYP3A5* genotype (data shown in Table 4), we could find that there was significant difference in Tmax of tacrolimus between *CYP3A5* expressers and *CYP3A5* non-expressers after taking WZC. The lower value of Tmax was seen in *CYP3A5* non-expressers (CYP3A5*3/*3) as well as lower V/F of tacrolimus in this group. When taking tacrolimus alone, there was significant difference of C_0_ between the 2 groups (*CYP3A5* expressers and non-expressers). As shown in Table 4, the value of C_0_ was higher in *CYP3A5* non-expressers than *CYP3A5* expressers.

**TABLE 3:** Pharmacokinetic parameters of tacrolimus with or without WZC with different *CYP3A5* genotype (*n* = 15).

Parameters		Genotype	CYP3A5*3/*3	p*	p**	p***	p****
		CYP3A5*1/*1 CYP3A5*1/*3					
t1/2	Tacrolimus alone	11.192 ± 8.774	11.628 ± 8.904	0.927	0.778	0.481	0.348
	with WZC	8.249 ± 5.842	7.406 ± 5.103				
Tmax	Tacrolimus alone	3.222 ± 2.108	2.667 ± 1.506	0.588	0.126	**0.004** ^**#**^	0.856
	with WZC	1.556 ± 1.014	2.500 ± 1.225				
V/F	Tacrolimus alone	375.739 ± 461.580	389.057 ± 367.487	0.954	0.309	0.235	0.102
	with WZC	131.908 ± 45.660	105.892 ± 45.167				
CL/F	Tacrolimus alone	24.421 ± 17.545	23.532 ± 14.192	0.919	0.752	**0.040** ^**#**^	0.093
	with WZC	12.864 ± 6.137	11.822 ± 5.734				
C_0_	Tacrolimus alone	2.401 ± 1.746	2.716 ± 1.423	0.9352	0.8584	0.0930	0.4916
	with WZC	4.631 ± 2.155	5.789 ± 2.759				
C_max_	Tacrolimus alone	5.743 ± 3.479	5.311 ± 2.739	0.7607	0.1359	0.0523	0.9086
	with WZC	16.420 ± 9.642	24.304 ± 14.709				

Note: Data are the mean ± SD. P* is considered to be the pharmacokinetic parameters comparison between the two groups when taking tacrolimus alone.

p** is considered to be the pharmacokinetic parameters comparison between the two groups when taking tacrolimus with WZC.

p*** is considered to be the comparison inside group A (CYP3A5*1/*1 or CYP3A5*1/*3) when tacrolimus was taken alone and with WZC.

P****is considered to be the comparison inside group B (CYP3A5*3/*3) when tacrolimus was taken alone and with WZC.

#*p* < 0.05 is considered to be statistically different.

**TABLE 4:** Pharmacokinetic parameters of tacrolimus with or without WZC in recipients with. different *CYP3A5* genotype (*n* = 15).

Parameters		Genotype	CYP3A5*3/*3	p*	p**	p***	p****
		CYP3A5*1/*1 CYP3A5*1/*3					
t1/2	Tacrolimus alone	13.219 ± 10.595	8.588 ± 2.747	0.252	0.682	0.327	0.400
	with WZC	8.401 ± 5.901	7.178 ± 4.937				
Tmax	Tacrolimus alone	3.556 ± 2.128	2.167 ± 0.983	0.160	**0.024** ^**#**^	0.149	0.111
	with WZC	2.444 ± 1.236	1.167 ± 0.408				
V/F	Tacrolimus alone	519.336 ± 493.223	173.661 ± 28.286	0.081	0.123	0.056	**0.012** ^**#**^
	with WZC	145.159 ± 65.553	104.936 ± 16.857				
CL/F	Tacrolimus alone	29.846 ± 17.980	15.395 ± 5.186	0.057	0.510	0.066	0.052
	with WZC	15.376 ± 10.701	12.243 ± 4.258				
C_0_	Tacrolimus alone	2.098 ± 1.388	3.033 ± 1.650	**0.046** ^**#**^	0.3253	0.0741	0.5758
	with WZC	4.765 ± 2.246	5.713 ± 2.042				
C_max_	Tacrolimus alone	4.645 ± 3.589	12.001 ± 10.189	0.2153	0.8025	0.0858	0.0919
	with WZC	19.258 ± 12.668	19.856 ± 9.988				

^#^ *p* < 0.05 is considered to be statistically different.

## 4 Discussion

Recently, the combination therapy of tacrolimus and WZC was common in clinical practice in China; however, besides the clinician experience, the related clinical study is indispensable. In addition, the comprehensive understanding of WZC mechanism on tacrolimus in liver transplant patients in the biomolecular aspect has rarely been investigated and remain elusive. Thus, it is essential to study the pharmacokinetics of both tacrolimus and WZC in liver transplant patients. Access to the accurate determination of blood concentration at different time points is the premise of conducting pharmacokinetics study. Therefore, in the present study, we developed and validated a sensitive and rapid liquid chromatography–tandem mass spectrometry (LC–MS/MS) method in multiple reaction monitoring (MRM) mode for simultaneous quantification of the tacrolimus and five bioactive lignan constituents (schisandrin, schisandrol B,schisantherin A, schisanhenol, and deoxyshisandrin) of WZC in human whole blood, and then applied this method to study the pharmacokinetics of all analytes in liver transplant patients. In addition, we first used this approach to further investigate the difference in tacrolimus pharmacokinetic parameters before and after taking WZC among different *CYP3A5* genotype donors and recipients.

To the best of our knowledge, it is the first time to report the simultaneous determination of tacrolimus, Deoxyshisandrin, schisandrin, schisantherin A, schisandrol B and schisanhenol in human whole blood and also the first time to study the pharmacokinetics of tacrolimus and WZC among different *CYP3A5* genotype donors and recipients. The pharmacokinetic parameters investigated here using our developed method was of great importance in our clinical practice, which might provide the reasonable basis for the interaction of the two drugs.

Several studies (Wang et al., 2016a; Kou et al., 2021) have reported that *Wuzhi* Capsule can improve the blood concentration of tacrolimus. Therefore, in clinical practice, liver transplant patients were often given WZC after taking tacrolimus to increase the blood concentration. A population PK model identifying tacrolimus daily dose, WZC daily dose, postoperative time, alanine transaminase, haemoglobin, total bilirubin, direct bilirubin, estimated glomerular filtration rate, and urea, concomitant with voriconazole and fluconazole was established in adult liver transplant patients (Du et al., 2022), the results of the increased blood concentration of tacrolimus in liver transplant patients were in line with our results. However, our study also investigated the genotypes of *CYP3A5* enzyme. Moreover, a PBPK analysis performed by He et al. also determined the increase of blood concentration of tacrolimus when co-administrated with WZC. Physiologically based pharmacokinetic (PBPK) modeling was one of the well-received strategies used to investigate the PK of drugs and DDI between drugs (Li et al., 2021). Compared with this, our currently constructed model had limitations in the terms of physiology information. Nonetheless, our individual PK model had the advantages of simple, model-independent characteristics. Consistent with one recent published paper, Lin et al. demonstrated that the blood concentration of tacrolimus could be increased with the combination of WZC without adverse effects (Yan et al., 2019). And our results partly validate the phenomenon. WZC could decrease the clearance of tacrolimus, slow down its elimination so as to increase its blood concentration. According to our study, there is an increasing trend in C_max_ and C_0_ of tacrolimus. However, there was no significantly statistical difference, which was not consistent with the previous study. We thought the difference might result from the poor sample size.

It is well known that tacrolimus is primarily metabolized by the *CYP3A5* in the liver and small intestine, of which partly contribute to the pharmacokinetics of tacrolimus. Given the well-documented influence of *CYP3A5* genotype on tacrolimus pharmacokinetics, the Clinical Pharmacogenetics Implementation Consortium (CPIC) recently published guidelines for *CYP3A5* genotype and tacrolimus dosing (K.A. Birdwell et al., 2015). As for the liver transplant patients, the guidelines suggest that the *CYP3A5* genotype of the recipient and donor could be determined. In conditions where the *CYP3A5* genotype is known, CPIC guidelines recommend increasing the initial dose of tacrolimus in *CYP3A5* expressers by 1.5–2 times the recommended starting dose, along with therapeutic drug monitoring to guide subsequent dose adjustments (K.A. Birdwell et al., 2015). It is also applicable to our present study. In our 15 Chinese liver transplant recipients, when taking tacrolimus alone, the C_0_ of *CYP3A5* non-expressers is obviously higher than *CYP3A5* expressers so they need lower doses of tacrolimus to reach target blood concentration, which is consistent with other studies (Cheng et al., 2015; Chen et al., 2017; Prytuła et al., 2019). What is new in our study is that we first studied the pharmacokinetics change of tacrolimus when combined with WZC among different *CYP3A5* genotype donors and recipients. Cheng et al.’s results also supported that *CYP3A5* genotype had significant influence in tacrolimus, whereas this was not observed in patients with other genotypes (Cheng et al., 2021). During our study, we divided the donors and recipients into two groups, respectively according to their genotypes. Then, we compared the tacrolimus pharmacokinetics of administration of TAC alone and co-administration with WZC within and between groups. At the initial of our study, we directly divided the patients (both donors and recipients) into three groups (wild type homozygous, heterozygous, mutation homozygous). Considering the small sample size of patients with CYP3A5*1/*1 (only two recipients in our study), we finally classified donors and recipients into two groups, in which we defined CYP3A5*1/*1 and CYP3A5*1/*3 as one group referring to other studies.

However, there still have some limitations in this study required for further improvement. First, the sample size in several genotypic groups in our study was small when patients were grouped according to different genotypes, which could influence the study results because of insufficient statistical power to some extent. What’s more, some other important factors and their complicated interactions that can affect tacrolimus disposition were not been considered in this study. Therefore, we will further optimize our study through a larger cohort in the future.

## 5 Conclusion

TDM is still a vital and necessary requirement in the use of tacrolimus. The method established here to simultaneously determine both tacrolimus and the main components of WZC would be critical for investigating the pharmacokinetics effects of WZC ontacrolimus. Though the determination of *CYP3A5* genotype may help achieve therapeutic tacrolimus concentration more rapidly with the co-administration of WZC, further validation is needed. What’s more, the relationship between *CYP3A5* genotype and postoperative acute rejection as well as adverse events also needs further study.

## Data Availability

The raw data supporting the conclusions of this article will be made available by the authors, without undue reservation.

## Abbreviations

WZC, Wuzhi Capsule; LC-MS/MS, liquid chromatography-tandem mass spectrometry; TDM, therapeutic drug monitoring; CYP, cytochrome P450; LLOQ, lower limit of quantification; QC, quality control; CPICI, Clinical Pharmacogenetics Implementation Consortium.
