# Supplementary material for: The pharmacokinetic study of tacrolimus and Wuzhi capsule in Chinese liver transplant patients
Source: Front Pharmacol. 2022 Sep 15;13:956166. doi: 10.3389/fphar.2022.956166 (PMC9520529; doi:10.3389/fphar.2022.956166)
Supplement: Supplementary file 1 [file DataSheet2.docx]

Supplementary Material

**This DOCX file includes:**

Supplementary Result

**Other supplementary materials for this manuscript includes:**

Additional file 1: Supplementary Method (Additional file 1.docx)

# Supplementary Results

**Detailed results for:**

**3.1 Method Validation**

The LC-MS/MS method in this study was previously developed and validated for simultaneous quantification of the tacrolimus and 5 bioactive lignan constituents (schisandrin, schisandrol B,schisantherin A, schisanhenol, and deoxyshisandrin ) in WZC in liver transplant patients (Wang et al., 2016). In our previous study of method establishment and validation, specificity results showed that there was no endogenous interference that displayed the same transitions or retention behavior as the analytes and IS in 6 different individual human blank whole blood samples. The response of any interfering peak with the same retention time as the analytes was less than 20% of the response of LLOQ (data not shown). Calibration curves were constructed via a linear-weighed least-squares (1/X^2^) regression method, and the linearity was good when the concentration of tacrolimus was in the range of 0.20–60 ng/ ml-, Schisantherin A 0.60-600ng /ml,Schisandrin 0.1-100ng/ ml, Schisandrol B 0.1-100ng/ ml, Deoxyshisandrin 0.1-100ng/ mlSchisanhenol 0.1-100ng /ml with coefficients r above 0.99 for all analytes. LLOQ of this analytical method was 0.2 ng/ml for tacrolimus, 0.6 ng/ml for schisantherin A and 0.1 ng/ml for the remaining components. Precision and accuracy of this method were tested with 6 replicates of QC samples at concentrations of low, medium, high and intra-day and inter-day precision was less than 10% for all quality control samples, and the accuracy was within the range of 85%-115% during the validation. In addition, all analytes could be extracted with extraction recoveries from 82.39% to 99.46% and matrix effects from 55.35% to 114.36%. Analytes stability was determined at three QC concentration levels in the aforementioned 4 conditions. The results of the three frozen-thaw cycle, short-term, long-term and processed sample stability studies revealed that all QC samples were found to be stable. All results indicated that the analytes fulfilled the validation parameters.

**References**

Wang, W., Zhu, S., Guo, W., Teng, F., Wei, H., and Chen, W. (2016). Simultaneous Determination of Tacrolimus and Five Main Compounds of Wuzhi Capsule in Liver Transplant Patients’ Whole Blood by LC-MS/MS. *Chin. J. Mod. Appl. Pharm.* 33, 854–859.
