# Supplementary material for: The pharmacokinetic study of tacrolimus and Wuzhi capsule in Chinese liver transplant patients
Source: Front Pharmacol. 2022 Sep 15;13:956166. doi: 10.3389/fphar.2022.956166 (PMC9520529; doi:10.3389/fphar.2022.956166)
Supplement: Supplementary file 2 [file DataSheet1.docx]

Supplementary Material

**This DOCX file includes:**

Supplementary Methods

**Other supplementary materials for this manuscript includes:**

Additional file 2: Supplementary Result (Additional file 2.docx)

# Supplementary Methods

**Detailed methods for:**

**2.5 Method Validation**

The validation was performed to evaluate the performance of the method based on the recommendations published by the FDA (US Food and Drug Administration, 2013). The selectivity and specificity of the method were established by evaluating 6 different batches of human blank whole blood samples and check was performed to avoid signals interfering with the signal of the analytes or the IS. Linearity of the calibration curve was confirmed by plotting the peak-area ratios of analytes to IS vs the analytes’ concentrations with 1/X^2^ weighted least-squared linear regression analysis. The lower limit of quantification (LLOQ) was obtained by analyzing 6 replicates of whole blood samples at the lowest concentration on the calibration curve. Intra-day and inter-day assays were performed on QC samples and measured in 6 replicates including low, medium and high concentration levels. Inter-day assays and accuracy were conducted on 3 consecutive days with calibration curves. Precision and accuracy were expressed as relative standard deviation (RSD) and relative error (RE), respectively. Recovery was determined at low, medium, high levels of QC samples. Three replicates of QC samples at each concentration level were analyzed by comparing the peak area ratios with those of the corresponding standards made post-extraction standards. The matrix effect of the analytes was determined by comparing the mean peak areas of the analytes in 3 replicates of post-extraction samples with those corresponding standards prepared by mobile phase. Stability was investigated in four different conditions for QC samples of 3 concentration levels in 3 replicates: 3 freeze-thaw cycles, room temperature (25℃) for 4 h for short term stability, 30 days frozen at -20℃ for long term stability, and 24 h for processed sample stability. See the detailed descriptions of these methods in our previous work (Wang et al., 2016).

**References**

Wang, W., Zhu, S., Guo, W., Teng, F., Wei, H., and Chen, W. (2016). Simultaneous Determination of Tacrolimus and Five Main Compounds of Wuzhi Capsule in Liver Transplant Patients’ Whole Blood by LC-MS/MS. *Chin. J. Mod. Appl. Pharm.* 33, 854–859.
